# Reconstruction of the Transmission History of RNA Virus Outbreaks Using Full Genome Sequences: Foot-and-Mouth Disease Virus in Bulgaria in 2011

**DOI:** 10.1371/journal.pone.0049650

**Published:** 2012-11-30

**Authors:** Begoña Valdazo-González, Lilyana Polihronova, Tsviatko Alexandrov, Preben Normann, Nick J. Knowles, Jef M. Hammond, Georgi K. Georgiev, Fuat Özyörük, Keith J. Sumption, Graham J. Belsham, Donald P. King

**Affiliations:** 1 Division of Livestock Viral Diseases, The Pirbright Institute, Pirbright, Surrey, United Kingdom; 2 Department of Exotic and Emerging Diseases, National Diagnostic and Research Veterinary Medical Institute, Sofia, Bulgaria; 3 Animal Health and Welfare Directorate, Bulgarian Food Safety Agency, Sofia, Bulgaria; 4 National Veterinary Institute, Technical University of Denmark, Lindholm, Kalvehave, Denmark; 5 Laboratory of Typing and Molecular Epidemiology, Foot-and-Mouth Disease Institute, Ankara, Turkey; 6 Animal Health Service, Animal Health Production and Health Division, Food and Agriculture Organization of the United Nations, Rome, Italy; Centers for Disease Control and Prevention, United States of America

## Abstract

Improvements to sequencing protocols and the development of computational phylogenetics have opened up opportunities to study the rapid evolution of RNA viruses in real time. In practical terms, these results can be combined with field data in order to reconstruct spatiotemporal scenarios that describe the origin and transmission pathways of viruses during an epidemic. In the case of notifiable diseases, such as foot-and-mouth disease (FMD), these analyses provide important insights into the epidemiology of field outbreaks that can support disease control programmes. This study reconstructs the origin and transmission history of the FMD outbreaks which occurred during 2011 in Burgas Province, Bulgaria, a country that had been previously FMD-free-without-vaccination since 1996. Nineteen full genome sequences (FGS) of FMD virus (FMDV) were generated and analysed, including eight representative viruses from all of the virus-positive outbreaks of the disease in the country and 11 closely-related contemporary viruses from countries in the region where FMD is endemic (Turkey and Israel). All Bulgarian sequences shared a single putative common ancestor which was closely related to the index case identified in wild boar. The closest relative from outside of Bulgaria was a FMDV collected during 2010 in Bursa (Anatolia, Turkey). Within Bulgaria, two discrete genetic clusters were detected that corresponded to two episodes of outbreaks that occurred during January and March-April 2011. The number of nucleotide substitutions that were present between, and within, these separate clusters provided evidence that undetected FMDV infection had occurred. These conclusions are supported by laboratory data that subsequently identified three additional FMDV-infected livestock premises by serosurveillance, as well as a number of antibody positive wild boar on both sides of the border with Turkish Thrace. This study highlights how FGS analysis can be used as an effective on-the-spot tool to support and help direct epidemiological investigations of field outbreaks.

## Introduction

Foot-and-mouth disease (FMD) is one of the most economically important animal diseases and notification of changes in disease status to the World Organisation for Animal Health (OIE) is compulsory. It is a highly contagious disease which spreads rapidly among susceptible cloven-hoofed animals (about 70 species) [1]. It hampers animal welfare and production in countries in where the disease is endemic within Asia and Africa, and parts of South America [2]. Sporadic incursions into disease-free areas can have a devastating socio-economic impact as typified by the epidemic in the United Kingdom (UK) in 2001, which resulted in approximately 2,000 infected premises, seven million slaughtered animals and £8 billion of direct and indirect losses [3].

The aetiological agent of FMD is FMD virus (FMDV) which belongs to the genus *Aphthovirus* in the family *Picornaviridae*. This is a non-enveloped, single-stranded positive-sense RNA virus with a rapidly evolving genome that is ca. 8200–8600 nucleotides (nt) in length. The FMDV genome consists of a long 5′ untranslated region (UTR) followed by a single open reading frame (ORF), a short 3′ UTR and a poly(A) tail [4]. The ORF is translated, under the control of an internal ribosome entry site, into a polyprotein which is eventually processed into 14 mature polypeptides: leader (L^pro^), 1A^cap^ (VP4), 1B^cap^ (VP2), 1C^cap^ (VP3), 1D^cap^ (VP1), 2A, 2B, 2C, 3A, 3B1^VPg1^, 3B2 ^VPg2^, 3B3 ^VPg3^, 3C^pro^ and 3D^pol^. The VPg peptide (of which there are three homologues) is covalently linked to the 5′ terminal uracil. Several factors contribute to the high evolutionary rate of FMDV including the high error-rate of the viral RNA polymerase (a characteristic shared with other RNA viruses) in combination with the fast rate of virus replication and large population size generated in infected cells [5]. These characteristics lead to the rapid fixation of mutations throughout the genome which are inherited by progeny viruses, a feature previously exploited to reconstruct transmission pathways of FMDV during field outbreaks of disease in the UK during 2001 [6]–[8] and 2007 [9]. In fact, FMDV has one of the highest synonymous substitution rates within animal RNA viruses, estimated at 8.29×10^−3^ substitutions/site/yr (confidence interval: 8.19–8.39×10^−3^) [10]. Genetic variability of FMDV, often classified on the basis of the sequences coding for the VP1 capsid protein of the virus (624–657 nt length), is reflected by the presence of seven antigenically distinct serotypes [O, A, C, Asia 1 and Southern African Territories (SAT) 1, SAT 2 and SAT 3] plus a large number of temporally and spatially distributed subgroups (topotypes, strains, lineages and sub-lineages) [2].

FMDV types O, A and more recently Asia 1, are endemic in the Anatolian region of Turkey and the Middle East. However, in Turkish Thrace only sporadic FMD outbreaks [in 1995, 1996 and 2007 involving the serotype O Middle East-South Asia (ME-SA) topotype, and in 2006 involving serotype A], have been reported in the last 25 years [11], [FAO World Reference Laboratory for FMD (WRLFMD) data]. Since 2010, Turkish Thrace has been recognised by the OIE as FMD free-with-vaccination. Despite the extensive efforts to control the disease within the region, FMD sometimes reaches neighbouring countries which are normally free-without vaccination [11]. This is the case for Bulgaria, which has experienced 3 limited outbreaks caused by strains within the O/ME-SA topotype in 1991, 1993 and 1996, but has otherwise been free from FMD since 1973 [12].

**Figure 1:**
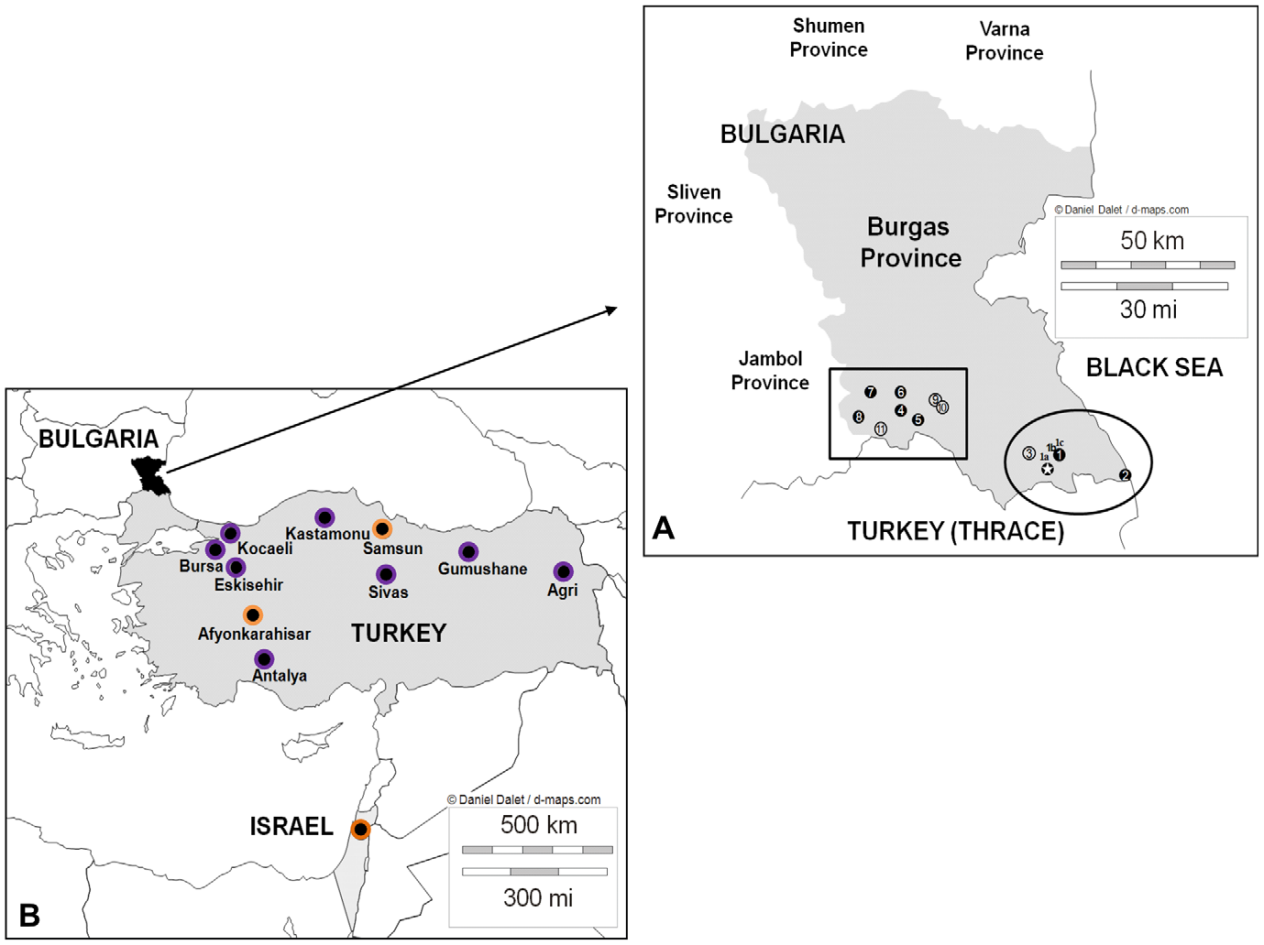
Location of FMDVs sequenced in this study. **A**) Map showing the locations of the outbreaks which occurred in Bulgaria during 2011. The two waves of outbreaks are delimited by a circle (1^st^ wave) and a rectangle (2^nd^ wave). Outbreaks are numbered and classified according to the virological status of the collected samples [FMDV-positive (•), and seropositive-only (○)]. The location of the FMDV-positive wild boar is highlighted with a white star. The 3 different sites in outbreak 1 are designated a to c. **B**) Map showing the sample location of the most closely related Turkish and Israeli FMDVs collected during 2010 (purple-surrounded circles) and 2011 (orange-surrounded circles) which were also included in the study.

On the 5^th^ January 2011, the Bulgarian authorities notified the OIE of the detection of FMDV RNA in samples collected from a wild boar with vesicular lesions on the feet. This animal had been hunted on the 30^th^ December 2010 close to Kosti village (Burgas region, Figure 1.) and the border with Turkish Thrace. Subsequent VP1 sequence analysis classified this virus as belonging to the ANT-10 sub-lineage of O/ME-SA/PanAsia-2, which had recently spread throughout the Middle East from Iran [13]. Moreover, this Bulgarian virus sequence differed by only one single nt, within the VP1 coding sequence, from sequences of contemporary FMDVs obtained from seven different Turkish provinces in Anatolia (Figure 1). The detection of FMDV in wild boar was followed by two waves involving a total of 11 FMD outbreaks in livestock within the same region (Table 1) over a 47 day period and up to 30 Km apart. The first wave (January 2011) included 3 outbreaks on at least five different sites. The second wave (March-April 2011), comprised eight outbreaks grouped in an area to the west of the first wave. FMD virus was recovered from seven outbreaks, two from the first and five from the second wave, whereas four outbreaks were only identified by serological survey. Approximately 2230 animals were slaughtered due to these disease outbreaks, whilst wider economic losses were incurred by countrywide restrictions on movement of animals and animal products, plus the negative impact on international trade due to the loss of FMD-free status.

**Table 1:** Summary of the FMDV outbreaks which occurred in Burgas, Bulgaria, 2011.

Designation	Date of confirmation	Location	Number of affected holdings	Number of affected/total animals	Maximum age of the lesions (days)	Viral RNA detection
Index	05/01/11	Kosti, Maketvetci, Tsarevo	-	1/1W	∼15	Yes
1	09/01/11	Kosti, Tsarevo	≥3^a^	1/194C, 14/117S, 12/149G, 8/72P	∼10 (1a); U(1b); 2–8 (1c)	Yes (1c only)
2	17/01/11	Rezovo, Tsarevo	1	3^b^/92C, 0^c^/77S&G	4/5–8	Yes
3	31/01/11	Gramatikovo, Malko Tarnovo	1	1/1C, 13/38S, 10/110G	U	No
4	19/03/11	Kirovo, Sredets	1	1^b^/143C	2–5	Yes
5	24/03/11	Granichar, Sredets	1	6/133C	5–8	Yes
6	25/03/11	Goliamo Bukovo, Sredets	1	4/49C, 0/11P	5–8	Yes
7	25/03/11	Fakia, Sredets	1	1/81C	3–5	Yes
8	25/03/11	Momina Tsarkva, Sredets	1	2/209C	3–5	Yes
9	03/04/11	Bliznak, Malko Tarnovo	Whole village	21/21C, 121/121S&G, 12/12P	U	No
10	03/04/11	Bliznak, Malko Tarnovo	1	121/121S&G, 72/72B	U	No
11	07/04/11	Dolno Yabalkovo, Sredets	Whole village	11/45C, 5/356S&G, 0/6P	U	No

W = Wild boar; C = Cattle; S = Sheep; G = Goat; P = Pig; B = Buffalo; U = Unobserved (FMDV-seropositive-only holding);

a 1a Seropositive free range pigs (lesions) and cattle; 1b Village with seropositive sheep, goats and pigs; 1c Virus positive Hereford cattle.

b Partial sampling.

c Clinical signs seen in sheep at culling.

This paper presents the results obtained using high resolution viral genome sequencing provided in real time during this outbreak. Full genome sequence (FGS) data was obtained for the FMDVs recovered during the outbreaks in Bulgaria 2011 (the virus detected in the wild boar plus one virus per virus-positive outbreak), plus 11 contemporary FMDVs from Turkey and Israel (collected during 2010 and 2011) which had VP1 sequences closest to the Bulgarian index case. These data were analysed using statistical parsimony and Bayesian Markov chain Monte Carlo (MCMC) inference and this information was combined with epidemiological data to reconstruct the spatiotemporal origin and transmission of the infection, as well as the molecular evolution of the virus during the outbreaks. These findings were compared with those obtained from similar studies on earlier FMD outbreaks [6], [7], [9], [14] to understand more fully the transmission dynamics of FMDV. These data highlight the importance and utility of FGS analysis when used in real-time to support FMD control programmes in the event of transboundary epidemics of disease.

## Materials and Methods

### Selection of viruses and FGS amplification and sequencing strategies

In total, 19 samples were selected for this study (Table 2). They were processed individually days apart and in parallel with a negative control to minimise and detect potential cross-contamination. Eight samples were derived from the Bulgarian outbreaks; one from the index case in wild boar with FMD lesions, and one from each of the seven FMDV-positive outbreaks in livestock. Within a virus-positive outbreak, the sample with strongest signal generated by FMDV real-time RT-PCR analysis was selected for sequencing (data not shown). The remaining 11 viruses came from Turkey (seven collected in 2010 and two in 2011) and Israel (one isolated in 2011). These viruses were selected for FGS analysis since VP1 sequence analysis demonstrated a close relationship with the viruses recovered from Bulgaria ([11]; unpublished data from WRLFMD). With the exception of a single sample from Israel (cell culture), all material tested was primary clinical material from epithelial lesions.

**Table 2:** Summary of full genome sequenced viruses generated in this study.

Virus Reference Number^a^		Host	Specimen^b^	Date collected	Origin (Outbreak/Location)	Sequencing^a^	Coverage	GenBank Accesion Number
WRLFMD	SAP/DTU							
BUL/1/1010	-	Wild boar	E	30/12/10	Bulgaria, Index case	WRLFMD	6.53	JX040485
-	12LPN1^c^	Cattle	E	14/01/11	Outbreak 1, Bulgaria	DTU	2.34	JX066664
-	12LPN3^c^	Cattle	E	16/01/11	Outbreak 2, Bulgaria	DTU	2.47	JX066665
BUL/11/2011	-	Cattle	E	18/03/11	Outbreak 4, Bulgaria	WRLFMD	5.71	JX040486
BUL/20/2011	-	Cattle	E	24/03/11	Outbreak 6, Bulgaria	WRLFMD	5.14	JX040487
BUL/26/2011	-	Cattle	E	23/03/11	Outbreak 5, Bulgaria	WRLFMD	4.28	JX040488
BUL/30/2011	-	Cattle	E	24/03/11	Outbreak 7, Bulgaria	WRLFMD	6.41	JX040489
BUL/32/2011	-	Cattle	E	24/03/11	Outbreak 8, Bulgaria	WRLFMD	7.40	JX040496
TUR/18/2010	-	Cattle	E	09/07/10	Kurtoğlu, Merkez, Gümü°hane, Turkey	WRLFMD	3.83	JX040491
^-^	TUR/840/2010^d^	Cattle	E	15/07/10	Asagikupkiran, Merkez, Agri, Turkey	WRLFMD	4.10	JX040493
^-^	TUR/868/2010^d^	Cattle	E	20/07/10	Sevinc, Odunpazari, Eskisehir, Turkey	WRLFMD	6.48	JX040494
^-^	TUR/883/2010^d^	Cattle	E	23/07/10	Kayi, Merkez, Kastamonu, Turkey	WRLFMD	5.38	JX040495
^-^	TUR/926/2010^d^	Cattle	E	26/07/10	Kozluca, Ýnegöl, Bursa, Turkey	WRLFMD	4.47	JX040496
^-^	TUR/1003/2010^d^	Cattle	E	10/08/10	Yavu, Yildizeli, Sivas, Turkey	WRLFMD	5.79	JX040497
TUR/36/2010	-	Cattle	E	13/08/10	Yenice, Izmit, Kocaeli, Turkey	WRLFMD	4.49	JX040492
^-^	TUR/1086/2010^d^	Cattle	E	16/08/10	Suleymaniye, Antalya, Turkey	WRLFMD	6.01	JX040498
TUR/8/2011	-	Cattle	E	14/01/11	Kirca, Sultandagi, Afyonkarahisar, Turkey	WRLFMD	6.40	JX040499
TUR27/2011	-	Cattle	E	01/01/11	Kozansiki,Kavak,Samsun, Turkey	WRLFMD	4.75	JX040500
ISR/2/2011	-	Cattle	CC	11/03/11	Kibbutz Bet Zera, Israel	WRLFMD	5.08	JX040501

a WRLFMD = World Laboratory Reference for Foot-and-Mouth Disease, The Pirbright Institute, United Kingdom; SAP = Foot-and-Mouth Disease Institute, Turkey; DTU = National Veterinary Institute, Denmark.

b E = Epithelium; CC = Cell culture.

c Sample nomenclature assigned by DTU.

d Sample nomenclature assigned by SAP.

Viral RNA was directly extracted from a 10% epithelium suspension (see preparation at [6]) or cell culture, and reverse-transcribed as previously described [15], [16]. After cDNA purification, complete FMDV genome sequences [except for the poly(C) region] were amplified and sequenced using a PCR strategy that generated 16 (at the National Veterinary Institute, Denmark) and 20 (4 additional redundant PCR reactions were performed as backup in case of potential reaction failure, at the World Laboratory Reference for Foot-and-Mouth Disease, UK) overlapping fragments of approximately 330 to 1400 base pairs. The primers (Tables S1 and S2) used have been described elsewhere [9], [15]–[18]. The negative control carried over from the RNA extraction step was used to check each PCR reaction and monitor the possibility of cross-contamination. Sequencing reactions were performed using Big Dye-Terminator v3.1 Cycle Sequencing Reaction Kit on an ABI 3730 DNA Analyzer (Applied Biosystems) following the manufacturer's instructions. Sequences were assembled, proof-read and edited with the Lasergene version 9.0 package (DNASTAR Inc, USA). These sequences have been submitted to GenBank and have been assigned the following accession numbers: JX040485-JX040501; JX066664-JX066665.

### Computational phylogenetic analysis

Alignment of the sequences was performed using BioEdit, Version 7.0.5.3 [19] and further manipulation and estimation of the dN/dS ratio was undertaken using DnaSP, Version 4.10.3 [20]. Maximum parsimony analyses were implemented in the TCS freeware, Version 1.21 [21]. Bayesian evolutionary analysis using Markov chain Monte Carlo (MCMC) sampling bases (30,000 trees from 30 million generations), as implemented using BEAST software, Version 1.6.1. ([22], http://beast.bio.ed.ac.uk), was carried out to estimate the rate of molecular evolution, to infer phylogenetic relationships and to implement the genetic spatiotemporal reconstruction of the outbreaks. Path-O-Gen Version 1.2 software (by A. Rambaut; http://tree.bio.ed.ac.uk/software/pathogen) was used to investigate the temporal signal and ‘clock-likeness’ of the data. Sampling collection dates and location were used to calibrate the molecular clock. jModelTest (Version 0.1.1) [23] was used to select the most appropriate nucleotide substitution model for these data. The robustness of the parameters was assessed by substituting different combinations of molecular clocks, demographic and phylogeographic diffusion models. The resulting spatiotemporal output was checked in Tracer, Version 1.5 and visualized with FigTree, Version 1.3.1. and GoogleEarth (http://earth.google.com) via Spread, Version 1.0.1. [24], as previously described [25].

## Results

### Nucleotide substitutions throughout the genome

Nineteen unique FMDV consensus sequences were generated for FMD viruses collected from Bulgaria, Turkey and Israel (Table 2). For all samples, the overlapping PCR strategy used yielded near complete genome sequences of 8170 nt in length with only 40–58 nt (0.51–0.71%) being derived directly from the primers used for the PCRs. The virus genome also includes an internal poly(C) tract (ca. 100–200 nt) within the 5′ UTR and a 3′ terminal poly(A) tail that were not included in this estimation. All of the component amplicons were of the expected size and no cross-contamination was detected within the negative control RT-PCR reactions which were performed in parallel. Nucleotide coverage ranged from 2.34 to 7.40 per site (Table 2) and no deletions or insertions were found in any of the genome sequences. A single ambiguity (A+G) in the large fragment of the 5′ UTR at nt position 844 [note, a 10 nt poly(C) sequence was included in the numbering system] was detected in the sequence obtained from the wild boar. This ambiguity was not considered for the statistical parsimony analysis.

**Figure 2:**
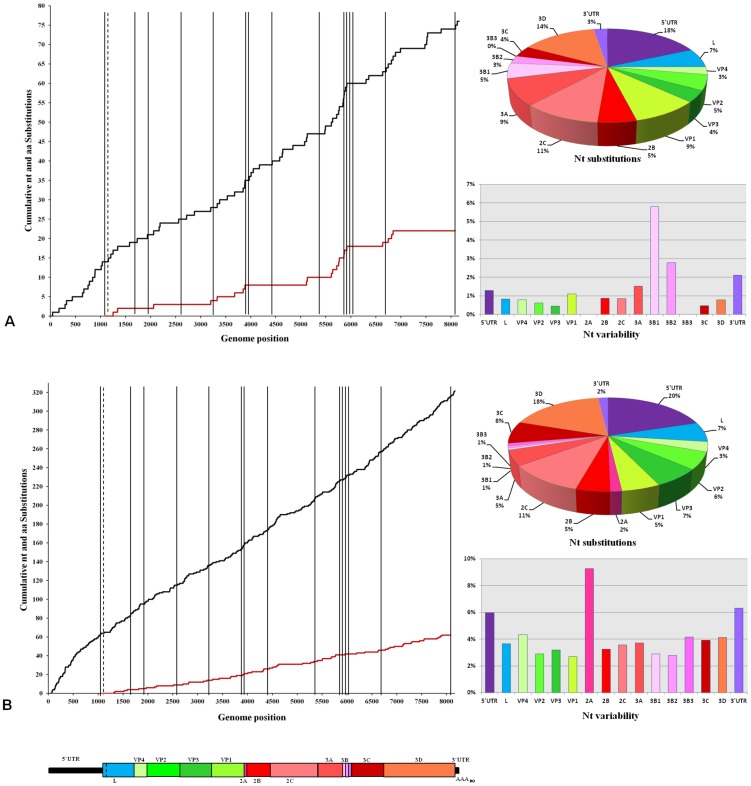
Nucleotide and amino acid substitutions occurring along the genome of the FMDV sequences. **A**) Data for sequences from Bulgaria (8 genomes): graphs represent the distribution of total nucleotide (nt) (black line) and non-synonymous (red) substitutions across the different genomic regions of FMDV (shown below). The pie chart and the bar chart show percentage of nt substitutions for each region, and nt variability within the region, respectively. **B**) Similar analysis to figure 2A) undertaken for the 11 FMDVs genomes from Turkey and Israel.

Comparison of the 19 sequences showed 415 nt substitutions at 413 different sites across the genome (Figures 2A and B). Positions 144 and 4581 (within the S-fragment of the 5′ UTR and the coding region for the 2C, respectively) presented three different nt variants. Of the nt substitutions in the open reading frame, 89 were non-synonymous changes while 233 were synonymous providing an overall non-synonymous to synonymous ratio (*d*N/*d*S) of 0.18. Codon 4090–4092 for the coding region for the 2B protein presented three different amino acid variants (asparagine – uncharged - to lysine and arginine – positive charge).

**Figure 3:**
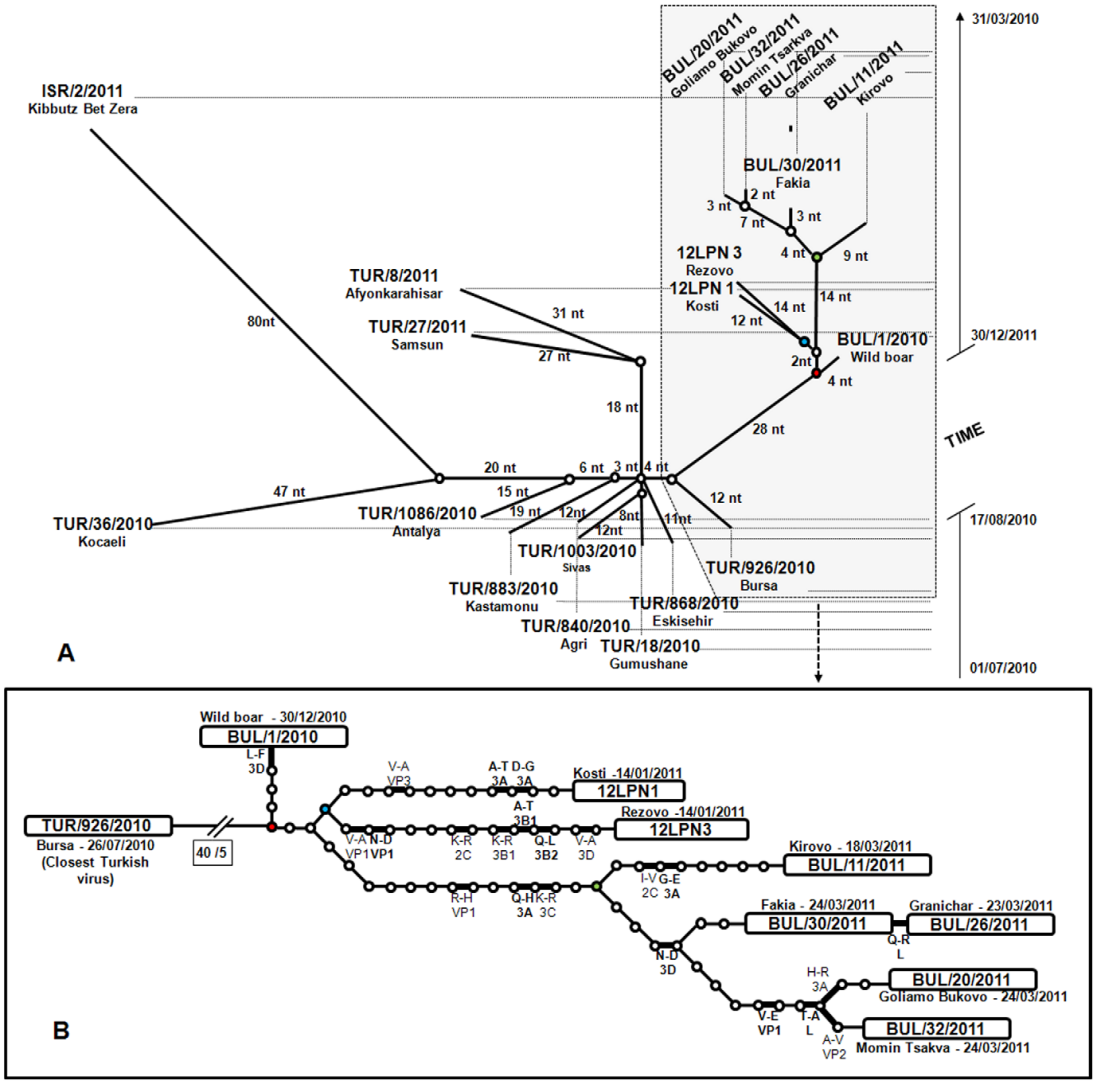
Statistical parsimony trees as implemented by TCS using the full genomes of 19 sequenced FMDVs. **A**. Edited TCS tree in which putative virus ancestors (○), except those corresponding to nodes, were removed. The length of the branches is directly proportional to the number of nucleotide (nt) changes. The vertical axis represents a time scale which denotes the date when the viruses were collected. **B**. Detailed TCS tree showing the viruses corresponding to the Bulgarian outbreaks and their closest ancestor within the Middle East. Open circles and lines correspond to putative genetic intermediates separated by single nt changes. Putative common (red circle) and secondary ancestors for each wave are shaded (blue circle, first; green circle, second). Lines in bold correspond to non-synonymous changes. The square shows the number of nt versus non-synonymous changes. The specific amino-acid changes are indicated, as well as the viral proteins involved. Non-conservative amino-acid substitutions (according to GONNET matrix, as implemented in BioEdit software) are highlighted in bold.

Substitution sites were distributed throughout the FMDV genome and provided greater resolution to distinguish field strains than relying on VP1 coding sequences alone, as apparent from previous studies [9]. Initial analysis highlighted seven Turkish FMDVs with identical VP1 coding sequences (of 633 nt) that differed by only one synonymous site to the sequence from the index case in wild boar [13]. However, using FGS analysis (8170 nt) the differences between these sequences and the first case in Bulgaria were expanded to between 44 and 60 nt (9–13 non-synonymous sites). Considering only the FMDVs collected from Bulgaria, a total of 75 nt substitutions at separate sites occurred along the genome (Figure 2A). Within the open-reading frame, 60 substitution sites were present of which 22 were non-synonymous changes (*d*N/*d*S = 0.23). Non-synonymous changes were distributed across structural and non-structural protein coding regions: 3A (five), VP1 (four), 3D (three); L, 2C, and 3B1 (two each): VP2, VP3, 3B2, 3C (one each). Sixteen out of the 22 non-synonymous changes were unique to individual isolates, while seven corresponded to a virus (BUL/12/LPN3/2011) recovered from the first phase of the outbreak in Bulgaria (Figure 3). Moreover, three out of these were non-conservative (in VP1, 3B1 and 3B2).

### Parsimony analysis (TCS)

Statistical parsimony analysis implemented by TCS [21] showed that all the sequenced Bulgarian viruses shared a unique putative common ancestor (Figure 3). The closest virus to this ancestor was BUL/1/2010, obtained from the wild boar hunted between Kosti and the border with Turkey on 30^th^ December 2010. Within the sequenced viruses from Turkey and Israel, TUR/926/2010, obtained in cattle from Bursa (Anatolia, Turkey) on 26^th^ July 2010, was the closest to the common ancestor of the Bulgarian viruses, differing by 40 nt which included five non-synonymous substitutions.

TCS analysis indicated that the virus obtained from the wild boar differed by only four nt, (including one non-synonymous substitution) from the putative common ancestor. The other Bulgarian sequenced viruses were derived from an intermediate ancestor from which two secondary putative ancestors, separated from each other by 15 nt (three non-synonymous) substitutions, seeded the two waves of outbreaks seen in livestock. The ancestor for the first wave was separated by only three nt substitutions from the Bulgarian common ancestor whereas the ancestor for the second wave was more distantly related (at 16 nt substitutions including three non-synonymous changes). Field viruses evolved on distinct branches (unique for each of the viruses from outbreaks 1, 2 and 4, i.e. BUL/12LPN1/2011, BUL/12LPN3/2011 and BUL/11/2011) from these secondary ancestors. The length of the branches ranged from seven to 14 nt changes (with two to seven being non-synonymous). Only two viruses collected at the end of the second wave (outbreaks 5 and 7, i.e. BUL/30/2011 and BUL/26/2011) were directly linked by a single nt (non-synonymous) difference.

### Bayesian analysis (BEAST)

Bayesian approaches as implemented with the BEAST software package [22]- were used to analyse the phylogenetic relationships between the viral sequences generated in this study. These results and interpretation (tree topology, evolution rates, and spatiotemporal intervals) were not exquisitely sensitive to the individual selected models and priors used in these analyses (data not shown). Furthermore, phylogenetic trees with similar topologies were also obtained by using Neighbor-joining methods (data not shown). Sequencing data was ‘clock-like’ except for the two isolates which were more genetically distant to the Bulgarian outbreaks (Figure S1). Estimation of the effective viral population size (Neτ, interpretable as the product of the effective number of infected animals and the virus generation time) through time using a Bayesian skyline plot (five potential population size transitions) did not show evidence of variation (Figure S2).

**Figure 4:**
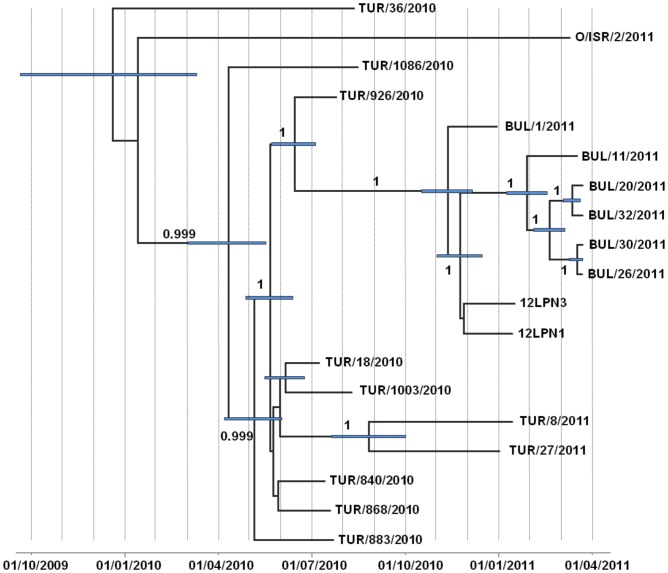
Bayesian maximum-clade-credibility time-scaled phylogenetic tree (BEAST) generated using 19 sequenced FMDV full genomes. The analysis was undertaken using a GTR substitution model, relaxed clock, constant population size, sampling 30.000 trees from 30 million generations. Uncertainty for the date of each node (95% highest posterior density – HPD - intervals) is displayed in bars. Only node labels with posterior over 0.8 are indicated. Overall, a rate of nucleotide substitution of 9.05×10^−3^ (95% HPD: 6.99–11.11×10^−3^) per site per year was estimated.

A relaxed molecular clock (GTR substitution model with no invariant sites, no heterogeneity of substitution rates among sites and a constant population; Figure 4) for all nt changes was found to advance at a rate of 9.05×10^−3^ substitutions per site per year (95% highest posterior density (HPD), 6.99–11.11×10^−3^). This timing reconstruction estimated that the putative common ancestor for the Bulgarian viruses was present from 11^th^ November 2010 (95% HPD: 17^th^ October to 5^th^ December, 2010) overlapping with the period of time estimated for the putative common ancestor of the outbreaks in livestock (23^th^ November 2010; 95% HPD: 1^st^ November to 15^th^ December, 2010). The estimated time for the secondary putative ancestor of the second wave is the 28^th^ January 2011 (95% HPD: 8^th^ January to 16^th^ February, 2011) whereas the intermediate between the Bulgarian sequences and the closest Turkish viruses was placed around the 15^th^ June 2010 (95% HPD: 23^th^ May to 5^th^ July 2010).

**Figure 5:**
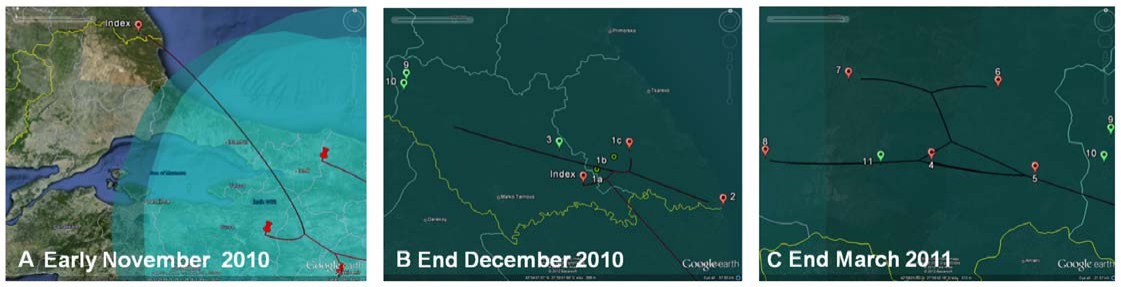
Spatiotemporal dynamics of the FMDV epidemic in Bulgarian 2011. The simplest option was considered for the selection of the gamma-random-relaxed-walk (RRW) continuous diffusion model. The isolate from Israel was excluded from this analysis,. Satellite imagery: GoogleEarth. Date accessed: 25 May 2012. Co-ordinates: 40°57′25.89″N, 28°27′28.38″E (A); 42°04′27.07″N, 27°39′47.60″E (B) 42°09′01.30″N, 27°09′42.18″E (C). **A**. FMDV spread from the North-West of Turkey throughout Bursa (Anatolia, Turkey) to Brugas (Bulgaria). The uncertainity on the location the virus is represented by transparent polygons (80% HPD). Turkish Thrace might have been infected before November 2010, which is plausible with the serological results in wild boar. **B**. The transmission infection pathways between the wild boar, outbreaks 1, 2, 3 and the second wave of outbreaks is not clarified. It might be explained by un-sampled notified sites/outbreaks or by a reservoir in wildlife (i.e. wild boar), both hypotheses are compatible with a genetically and spatiotemporally close FMDV replicating within a host. **C**. The genetic spatiotemporal reconstruction of the second wave of outbreaks linked them to each other, in agreement with epidemiological data, i.e. owners from animals in outbreak 6 had animals in the location of outbreak 4.

A gamma-random-relaxed-walk (RRW) continuous diffusion model was selected to reconstruct the incursion of FMDV into Bulgaria from Anatolia (comparison data with other RRW models not shown). The results are shown in Video S1 and S2 [Satellite imagery: GoogleEarth. Data accessed: 25 May 2012. Co-ordinates: 45°55′15.46″N, 22°57′21.60″E (Video S1); 42°21′43.62″N, 26°59′08.64″E (Video S2)] and summarized in Figure 5. The shared viral ancestors of the Bulgarian wild boar and all the viruses in livestock are located close to the place where the wild boar was shot and outbreak 1 (sites a, b and c) near Kosti whereas the immediate ancestor of the second wave is spatially close to the second cluster of outbreaks. Spatial uncertainty (80% HPD region) highlighted the Turkish Thrace since April 2010 and all the Burgas region since early November 2010.

### Links between laboratory, genetic and epidemiological field data

**Figure 6:**
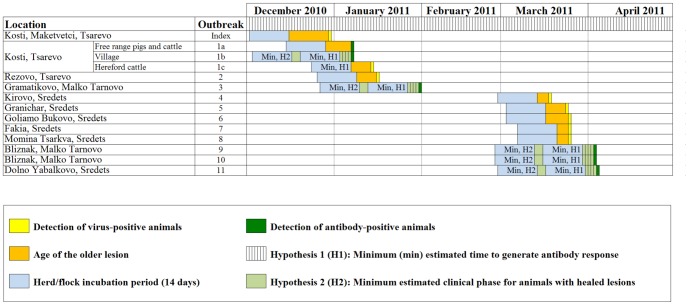
Estimated time in which FMDV might have been introduced causing the different Bulgarian outbreaks. This illustration was built according to the date of sample collection, the virus and serological results of the collected samples and the age of the lesions of the animals with clinical signs. The date of sample collection is coloured in yellow in case of the virus-positive outbreaks, and in dark green in case of the seropositive-only outbreaks. The age of the lesions of the animals with clinical signs (if any), according to the National Veterinary surgeons involved in the outbreaks, is coloured in orange. The incubation time, estimated to be 14 days, is coloured in blue. In the case of the seropositive-only outbreaks, two different times were considered to explain the presence of antibody-positive/virus-negative samples, depending on whether clinical signs where unobserved or mild (hypothesis 1, H1) or whether the lesions had healed (H2). In case of H1, a minimum of 5 days post-infection was estimated (shaded with vertical stripes), whereas a minimum of 21 days post-infection for H2 (pale green). All estimated times were based on previous studies [1], [9]. Only genetic data can prove a link between waves one and two.

Sero-surveillance using at least one of the established diagnostic ELISAs that detect antibodies against the structural (Priocheck FMDV type O ELISA, Prionics, Lelystad B.V) or non-structural (Chekit FMD-3ABC bo-ov Antibody Test Kit, IDEXX; Priocheck FMDV NS ELISA, Prionics, Lelystad B.V) proteins of FMDV provided evidence that FMDV infection had occurred on additional livestock premises where clinical disease (and hence viral RNA) had not been observed/detected (Table 1, Figure 1). These premises included two additional sites, considered within outbreak 1 and infected earlier than the herd of Hereford cattle from which the FMDV sample was obtained and sequenced (1c): 1a, comprising free-range pigs and cattle, including one pig with healed FMD lesions; and 1b, the village of Kosti including about 400 susceptible livestock. Similarly, antibodies but no FMD viral RNA were detected from outbreaks 3 (first wave), 9, 10 and 11 (second wave). Moreover, all animals from outbreaks 9 and 10 were seropositive, including 12 pigs (Table 1). In order to place these missing viruses within the transmission pathways for the outbreaks, a temporal epidemiological reconstruction was carried out to estimate the potential infection date for each of the different sites/outbreaks (Figure 6). This reconstruction was based on the age of the lesions when they were reported. When no reports existed (such as for the antibody-positive/virus-negative outbreaks), lesions were considered to be: H1, mild or overlooked, therefore a minimum of 5 days post-infection was estimated for the antibodies to have appeared and to clear the virus [26]; or H2, healed, therefore a minimum of 21 days post-infection was estimated [9]. In the case of outbreak 11, H1 is more likely because of the low sero-prevalence within the animals and the fact that they had been tested as part of the surveillance activities four times since the start of the outbreak, the last time was 14 days before being found to be virus-positive. In contrast, H2 is the most plausible option to explain the 100% seroprevalence within outbreaks 9 and 10. Figure 6 supports the genetic spatiotemporal reconstruction of the origin and transmission of the infection in Burgas region, highlighting the role played by only-seropositive sites/outbreaks in livestock in the length of the branches of the genetic tree.

## Discussion

Using full FMDV genome sequences from clinical samples it has been possible to reconstruct the likely origins and transmission pathways within FMDV outbreaks in the UK at high resolution [6]–[9]. These data can be used to support known patterns of spread of the virus [6], as well as to reveal links that are not apparent or different from those suggested by epidemiological investigation at the field level [7]–[9]. Sequence data alone cannot define the specific sources of infection and modes of transmission between outbreaks. However, analyses of these data can provide insights into the way that the infection has spread between farms and substantiate the temporal dynamics of an epidemic. This approach was particularly appropriate for the FMDV outbreaks in Bulgaria during 2011, since there were no clear epidemiological data that could be used to pin-point the origin of these outbreaks, or to define the transmission links between the two spatially distinct waves that were observed during January and March/April.

The Bulgarian viruses were all very closely related to contemporary FMDVs from the Middle East, particularly seven viruses collected from Anatolia (Turkey) during summer 2010 (≥99.2% identity, complete genome). Of these seven viruses, the most closely related sequence differing by 44 nt substitutions was a FMDV collected on the 26^th^ July 2010 in Bursa. Although the data is consistent with a source from Turkey, our analyses using TCS and Bayesian methods suggest that the immediate ancestor and direct source of the outbreaks in Bulgaria has not been sampled. Based on rates of nt substitution observed during other field outbreaks [6], an average rate of 8.9×10^−3^ (HDP: 8.5–11.3×10^−3^) rate between the closest Turkish sequence and the putative common ancestor in Bulgaria are compatible with continuous viral replication within susceptible hosts. Interestingly, the FMDVs collected in Anatolia at the beginning of 2011 were more distantly related (≤99% identity) to the index case in Bulgaria than the seven FMD viruses collected in 2010.

The presence of a single putative common ancestor around November/December 2010 for all the sequences recovered from infected animals in Bulgaria provides clear evidence for a single introduction of the virus. The timing of this ancestor coincides with the sacrifice day of the Kurban Bayram Festival (16^th^ November 2010) which is associated with increasing risk of illegal practices and, potentially, increased disposal of meat/offal for the access of wild animals. The index case in wild boar, hunted near the border with Turkish Thrace at the end of December 2010, provided the sequence which was most closely related to the closest ancestor of all of the Bulgarian outbreaks in livestock. Wild boars (*Sus scrofa*) are omnivorous and social animals, susceptible to FMDV [27], which are native to Bulgaria and many other countries within Europe and Asia. Their density in Europe is low but they are able to travel long distances. Serological results for the occurrence of FMDV infection in wild boar from the Thrace region of Turkey carried out after the outbreaks in Bulgaria [28] provided evidence that FMDV has also infected wild boar in this transboundary region. Experimental studies using feral pigs [29] or wild boar [28] indicate that clinical signs of FMD infection in these hosts can be milder and that the incubation period can be longer than in domesticated pigs. Moreover, these studies have indicated that, at least under these close contact conditions, wild boar can transmit the virus to domestic pigs. However, the situation in the “field” could be rather different. In the case of the outbreaks in Bulgaria in 2011, there are reports (EU Veterinary Expert Team) of direct contact between wild boar and domesticated livestock from site 1a (outbreak 1). Accordingly, it is unclear which animal species were infected first.

One important aspect of FGS analyses is the use of the number of farm-to-farm nucleotide changes to predict the presence of un-sampled cases that contribute to the epidemiology of an endemic, as carried out during the 2007 outbreak in UK [9]. The TCS analyses of the Bulgarian sequences carried out in real-time highlighted a number of long branches (more than 10 nt substitutions) between the common ancestor and viruses collected from outbreaks 1 (Kosti), 2 (Rezovo) and the series of outbreaks within the second wave. The presence of undetected infection within susceptible animals were confirmed by subsequent serosurveys in the farms of the reported outbreaks where FMDV was not recovered: Gramatikovo (outbreak 3), Bliznak (outbreaks 9 and 10), and Dolno Yabalkovo (outbreak 11). Serological evidence for FMDV infection was also obtained for locations 1a and 1b within outbreak 1 (Kosti). It is not possible to pin-point confidently the position of un-sampled FMDVs on the TCS tree. Moreover, they might be genetically distinct from those described in the other outbreaks. However, it is likely that viruses from seropositive-only farms are placed within the long branches present on the TCS tree. With this in mind, the reconstruction of a plausible transmission pathway for the outbreaks in domesticated livestock in Bulgaria would be as follows: the first wave comprising initial infection on outbreak 1 (1a or 1b, most likely, and from there to 1c) that spread to outbreak 2 and 3. Transmission of FMDV from outbreak 2 to outbreak 3 is less likely, because of geographical reasons. The second phase of outbreaks is more complex: probably seeded from outbreak 1 and with the possible involvement of outbreak 3, it also probably involved infection through intermediate outbreaks 9 and 10, or less likely outbreak 11 (Dolno Yabalkovo), where animals were seronegative 14 days prior to being declared seropositive. Of the subsequent outbreaks, only viruses from outbreak 7 to outbreak 5 are directly linked (single nt change) indicating local spread.

From an epidemiological perspective, the FMDV outbreaks in Bulgaria share some similarities to those that occurred during 2007 in the UK. Both affected a small number of premises in two waves without clear epidemiological links and separated by time and space [9]. However, the TCS tree obtained with the viruses obtained from the outbreaks in the UK was a linear tree, in contrast to the branched TCS tree obtained from the Bulgarian sequences. Direct extrapolation of parameters between these two studies should be attempted cautiously because there are a number of key differences that may have increased the number of observed nt substitutions that accrued during farm-to-farm (or outbreak-to-outbreak) transmission. Of these factors, the most important differences to the previously described FMD outbreaks in the UK are: i) the larger (approximately four times) number of infected animals in Bulgaria; ii) the extensive nature (in a forested area) of livestock management; iii) lower frequency of owner-inspections; and iv) the focus of virus diagnostic testing towards only cattle and buffalo. In addition, some of the FMD cases in Bulgaria also involved pigs (outbreaks 1 and 9, included eight and 12 infected pigs, respectively), which can release high levels of viruses [1]. Therefore, further studies are required to determine whether non-sampled viruses from the seropositive-only outbreaks within domestic animals are enough to explain the length of the branches obtained when analysing the sequences of the Bulgarian outbreaks, or whether wildlife (wild boar) contributed to the transmission pathways during these outbreaks.

Although it is well known that FMDV can infect a wide range of different species, the epidemiological significance of wildlife for maintenance of virus circulation is not well established with the exception of African buffalo (*Syncerus caffer*) which maintain the virus within herds for many years [30]. Within Europe, the widespread outbreaks of FMDV which occurred until the early 1980s were controlled just within the domestic animal population, and when spread occurred to wildlife, the disease was not maintained. However, since the cessation of vaccination against FMDV in Europe (in the early 1990s), which lead to all cloven-hoofed animals becoming susceptible, there has been a significant increase in the population of some susceptible wild life species, such as wild boar [28]. The fact that the initial case of FMDV in Bulgaria, declared in January 2011, was in wild boar suggested, for the first time, that such susceptible wildlife could pose a threat to domestic animals. It is clearly important to establish if wildlife, within Europe, can maintain virus circulation and whether they represent a significant risk for domestic animals or whether the wildlife are only infected by “spill over” from outbreaks in domestic species. Further surveillance is warranted to define the contribution and risks associated with wildlife.

Although the precise mechanisms by which FMDV was transmitted between the infected farms are more difficult to pin-point, these analyses provide useful insights into the relationship between the viruses which were present in Bulgaria. In fact, the potential existence of undisclosed infection was reported in real-time to the EU Mission and the National Veterinary Authorities in Bulgaria: findings that were confirmed later by serology (different sites/holdings in outbreak 1 and outbreak 3 within the first wave; outbreaks 9, 10 and 11, from the second wave). This study highlights how full genome sequence analysis of a rapidly evolving RNA virus can be used as a real-time tool to support and help direct epidemiological investigations of outbreaks of transboundary diseases caused by RNA viruses.

## Conclusions

The genetic diversity of FMDV within a limited epidemic in the region of Burgas (Bulgaria, an FMD-free-without-vaccination country) in early 2011 of unknown origin was used in real-time to the support the FMD control policy. Statistical parsimony methods and Bayesian Markov chain Monte Carlo inference of the sequences were used to reconstruct the spatiotemporal origin and transmission of these outbreaks. These data disclosed undetected infection, in livestock and/or in wildlife (wild boar), and linked the two different waves of outbreaks within the region. This study highlights the practical approach of combining complete genome sequencing, computational phylogenetics and epidemiological field data to understand the spread of RNA viruses and transboundary diseases.

## Supporting Information

Figure S1 **Evaluation of the temporal signal and ‘clock-likeness’ of the data (Path-O-Gen).** **A**. Regression of root-to-tip distances against date of sampling of 19 sequences to investigate the ‘clock-likeness’ of its molecular phylogeny. **B**. ‘Best fitting root’ to the hypothesis that the 19 viruses have a constant rate of evolution.
(TIF)

Figure S2 **Estimation of viral effective population size (Neτ, interpretable as the product of the effective number of infected animals and the virus generation time) through time using a Bayesian skyline plot (BEAST).** Five potential population size transitions underlay the demographic model. The thick solid black line is the median estimate and the blue lines show the 95% HPD limits. The dashed lines (and shadowed areas) represent the estimated time (with 95% HPD) of the ancestor of (from left to right): 1. The closest of the Turkish viruses and the Bulgarian viruses; 2. The Bulgarian viruses; 3. The Bulgarian viruses in livestock; 4. the second wave of Bulgarian viruses.
(TIF)

Table S1 **Oligonucleotide primers used for the FMDV amplification and sequencing at The Pirbright Institute (UK).**
(DOCX)

Table S2 **Oligonucleotide primers used for the FMDV amplification and sequencing at the National Veterinary Institute (Denmark).**
(DOCX)

Video S1 **Spatiotemporal reconstruction of the incursion of FMDV into Bulgaria from Anatolia (Turkey).**
(MP4)

Video S2 **Spatiotemporal reconstruction of the incursion of FMDV within Burgas region (Bulgaria).**
(MP4)
