# Supplementary figures and images for: Reconstruction of the Transmission History of RNA Virus Outbreaks Using Full Genome Sequences: Foot-and-Mouth Disease Virus in Bulgaria in 2011
Source: PLoS One. 2012 Nov 30;7(11):e49650. doi: 10.1371/journal.pone.0049650 (PMC3511503; doi:10.1371/journal.pone.0049650)

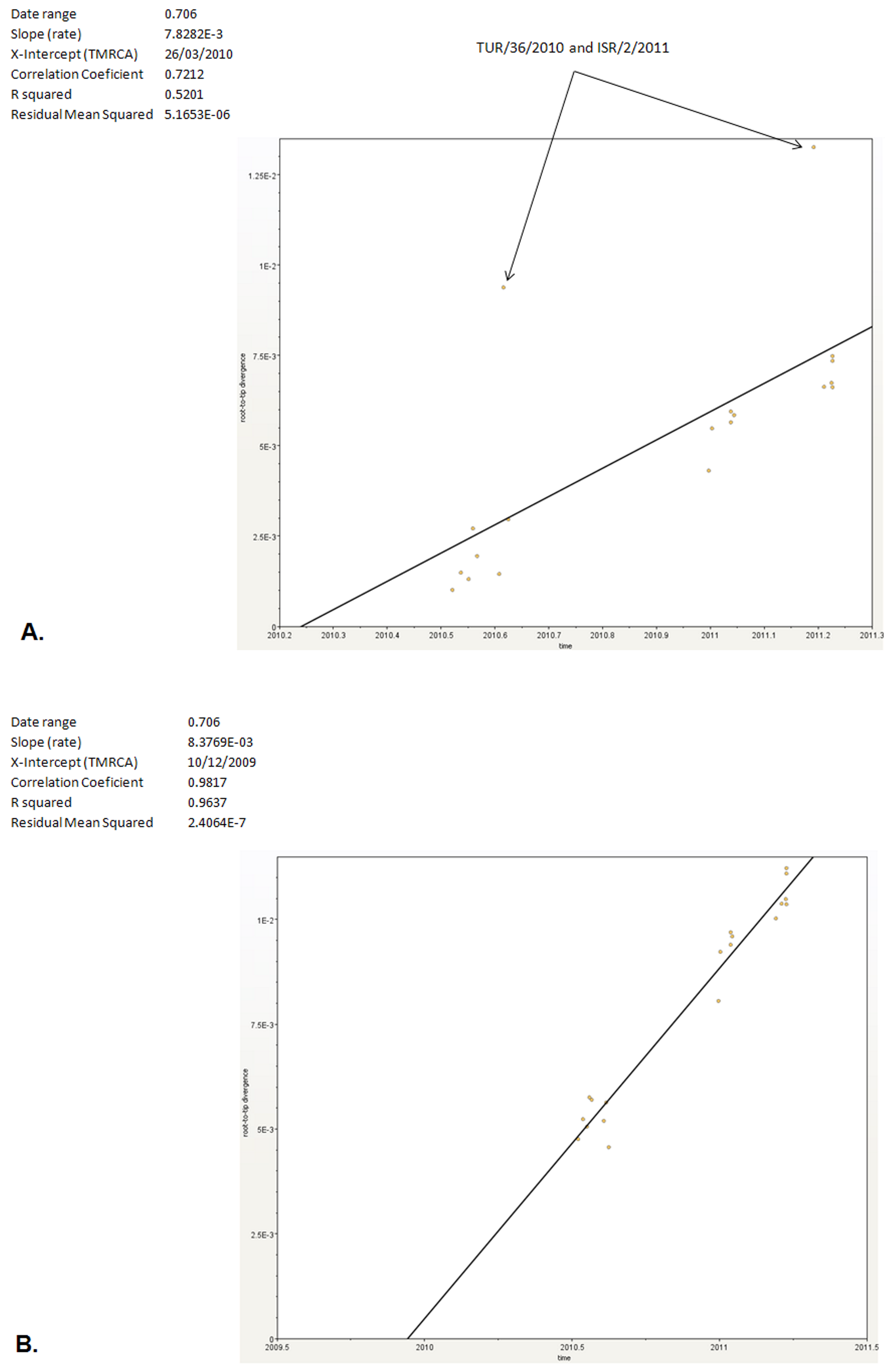

Supplement: Figure S1 — Evaluation of the temporal signal and ‘clock-likeness’ of the data (Path-O-Gen). A. Regression of root-to-tip distances against date of sampling of 19 sequences to investigate the ‘clock-likeness’ of its molecular phylogeny. B. ‘Best fitting root’ to the hypothesis that the 19 viruses have a constant rate of evolution. (TIF) [file pone.0049650.s001.tif]

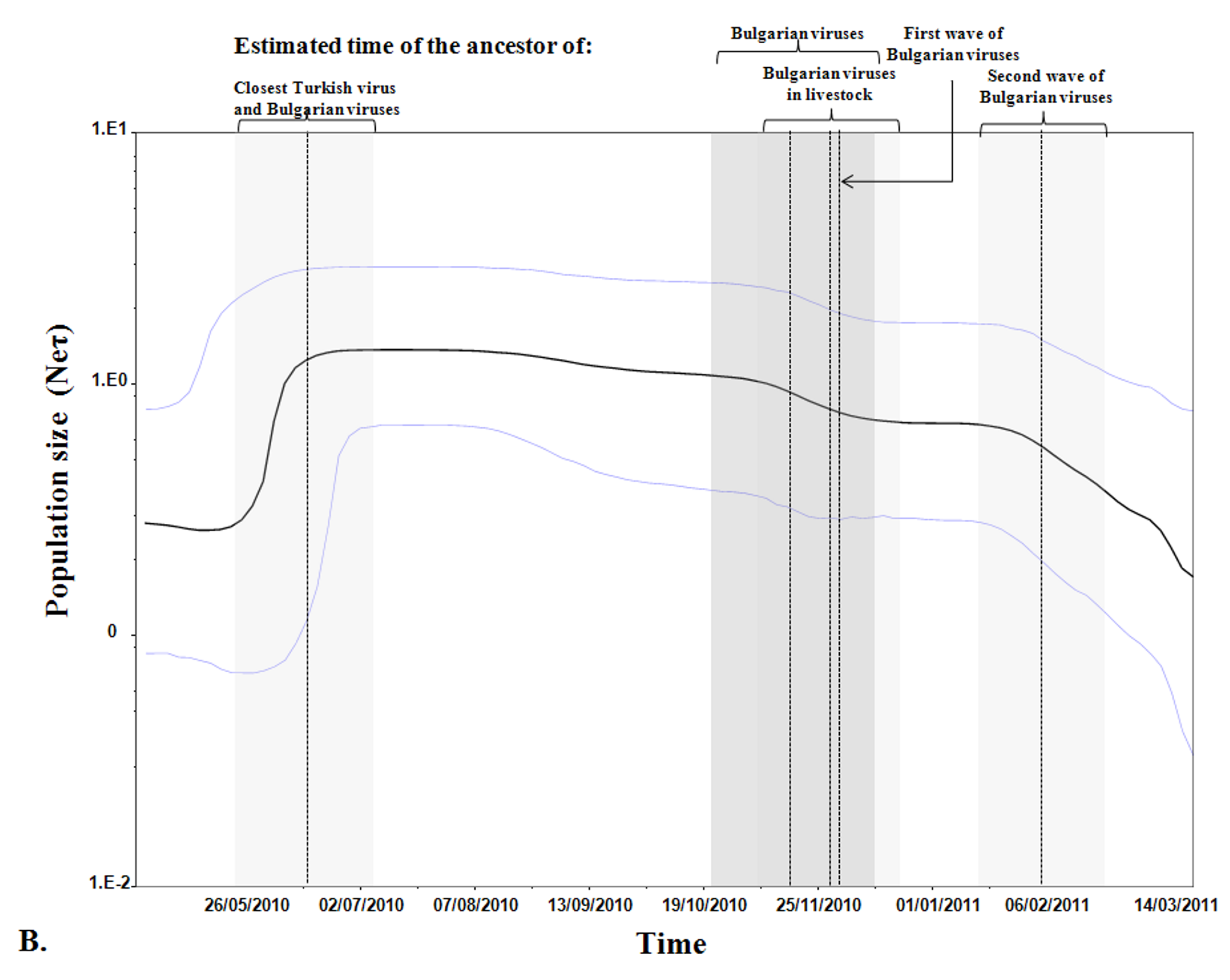

Supplement: Figure S2 — Estimation of viral effective population size (Neτ, interpretable as the product of the effective number of infected animals and the virus generation time) through time using a Bayesian skyline plot (BEAST). Five potential population size transitions underlay the demographic model. The thick solid black line is the median estimate and the blue lines show the 95% HPD limits. The dashed lines (and shadowed areas) represent the estimated time (with 95% HPD) of the ancestor of (from left to right): 1. The closest of the Turkish viruses and the Bulgarian viruses; 2. The Bulgarian viruses; 3. The Bulgarian viruses in livestock; 4. the second wave of Bulgarian viruses. (TIF) [file pone.0049650.s002.tif]
