# Supplementary material for: Reconstruction of the Transmission History of RNA Virus Outbreaks Using Full Genome Sequences: Foot-and-Mouth Disease Virus in Bulgaria in 2011
Source: PLoS One. 2012 Nov 30;7(11):e49650. doi: 10.1371/journal.pone.0049650 (PMC3511503; doi:10.1371/journal.pone.0049650)
Supplement: Table S1 — Oligonucleotide primers used for the FMDV amplification and sequencing at The Pirbright Institute (UK). (DOCX) [file pone.0049650.s003.docx]

| **Fragment** | **Primer name** | **Primer Sequence 5’ – 3’** | **Reference** |
| --- | --- | --- | --- |
| 1 | O1F | TTGAAAGGGGGCGYTAGGGTYTCA | 1 |
|  | O1R | CRAAGCCYRCCTTTCACCC | 1 |
| 2 | BFS-370F | CCCCCCCCCCCCCTAAG | 2 |
|  | OP2-1633R | AGAACGTCGGACGGGTCC | This work |
| 3 | O3F | GTGTGCAACCCCAGCACG | 1 |
|  | O4R2 | CGGTTTTCTTGTCGGCGAG | 1 |
| 4 | O4F | GTGGACCACCCGCTCTC | 1 |
|  | O5R | TCTGGCACCATGGCCAC | 1 |
| 5 | O5F | CAGAACCARTCAGGCAACACTG | 1 |
|  | O6R2 | GCCTCAGCCACATCAAGG | 1 |
| 6 | O6F | CTGACCACAAAGGTGTCTAYGG | 1 |
|  | O7R | GTCAGACGCGGTGTACGC | 1 |
| 7 | O7F | CACGTYGCGGGTGAGTTCC | 1 |
|  | O8R | CGGGACCCAGGTRAGGTTYC | 1 |
| 8 | O8F | CACACAGTACAGCGGCACC | 1 |
|  | 09R | CCCTCTTCATGCGGTAAAGC | 1 |
| 9 | O9F | GCTGACCCCGTGACYGC | 1 |
|  | OP2-4199R | ACCGTGCTGCTACAGCGG | This work |
| 10 | O10F | CTTGCACTGCCTTACACGGC | 1 |
|  | PCR NSP 1R2 | CTTCTGAGGCGATCCATG | 1 |
| 11 | PCR NSP 1F | GAGACGTYGAGTCCAACCC | 1 |
|  | PCR NSP 2R | GCCATRGGCGGGATRAA | 1 |
| 12 | PCR NSP 2F | CAGCTCARAGCACGTGACAT | 1 |
|  | PCR NSP 3R | ACCATCCCCTCRAAGAAYTC | 1 |
| 13 | PCR NSP 3F | TGACCACTTYGACGGTTA | 1 |
|  | PCR NSP 4R | CATRATCACTATGTTTGCCA | 1 |
| 14 | PCR NSP 4F | CGRAGGTTYCACTTTGAC | 1 |
|  | PCR NSP 5R | CACTTTCAAAGCGACAGG | 1 |
| 15 | PCR NSP 5F | GAATTCTTTGAGGGGATGGT | 1 |
|  | PCR NSP 6R | GGGGGTKCCYTTCTTCAT | 1 |
| 16 | PCR NSP 6F | CRAGCTGAAGGACCCTAC | 1 |
|  | PCR NSP 7R | GACGCGTAGTCRGCAGC | 1 |
| 17 | PCR NSP 7F | GGACAGGACATGCTCTCAG | 1 |
|  | PCR NSP 8R | AATTTGCGGTCCGTTGT | 1 |
| 18 | PCR NSP 8F | ATGCGCAAAACCAAGCT | 1 |
|  | PCR NSP 9R | GTCCAGCTCRACTCCCTC | 1 |
| 19 | PCR NSP 9F | RACCTTCCTGAAGGACGAR | 1 |
|  | PCR NSP 10R | ACTTCTCCTGTATGGTCCCA | 1 |
| 20 | PCR NSP 10F | AACGTGTGGGATGTGGA | 1 |
|  | RACE-T21G | CAGGAAACAGCTATGACTTTTTTTTTTTTTTTTTTTTTG | 1 |
| 21 | PCR NSP 10F | AACGTGTGGGATGTGGA | 1 |
|  | RACE-T21C | CAGGAAACAGCTATGACTTTTTTTTTTTTTTTTTTTTTC | 1 |
| 22 | BFS-1F | TTGAAAGGGGGCRCTAG | 2 |
|  | BFS-379R | GGGGGGGGGGGGTGAAA | 2 |
| 23 | A-380F | CCCTAAGTTTTACCGTCRYWC CCG | This work |
|  | O4R2 | CGGTTTTCTTGTCGGCGAG | 1 |
| 24 | O7F | CACGTYGCGGGTGAGTTCC | 1 |
|  | 09R | CCCTCTTCATGCGGTAAAGC | 1 |

**Table S1 References**

**1:** Abdul-Hamid NF, Firat-Sarac M, Radford AD, Knowles NJ, King DP (2011) Comparative sequence analysis of representative foot-and-mouth disease virus genomes from Southeast Asia. Virus Genes 43: 41-45.

**2:** Cottam EM, Wadsworth J, Shaw AE, Rowlands RJ, Goatley L, et al. (2008) Transmission pathways of foot-and-mouth disease virus in the United Kingdom in 2007. PLoS Pathog. 2008 Apr 18;4(4):e1000050.
