# Supplementary material for: Reconstruction of the Transmission History of RNA Virus Outbreaks Using Full Genome Sequences: Foot-and-Mouth Disease Virus in Bulgaria in 2011
Source: PLoS One. 2012 Nov 30;7(11):e49650. doi: 10.1371/journal.pone.0049650 (PMC3511503; doi:10.1371/journal.pone.0049650)
Supplement: Table S2 — Oligonucleotide primers used for the FMDV amplification and sequencing at the National Veterinary Institute (Denmark). (DOCX) [file pone.0049650.s004.docx]

| **Fragment** | **Primername** | **Primer Sequence 5’ – 3’** | **Reference** |
| --- | --- | --- | --- |
| 1 | O1F | TTGAAAGGGGGCGYTAGGGTYTCA | 2 |
|  | O1R | CRAAGCCYRCCTTTCACCC | 2 |
| 2 | BFS-370F | CCCCCCCCCCCCCTAAG | 2 |
|  | 8-A PN 63 | AGACCTGGAAAGACCAGGC | This work |
| 3 | 8-A PN 35 | GAGAAAIGGGACGTCIGCGC | 1,4 |
|  | 8-A PN 2 | GTCICCTATTCAGGCITAGAAG | 1,4 |
| 4 | 8-A PN 3 | GGCTAAGGATGCCCTTCAG | 1,4 |
|  | 8-A PN 4 | AACCAGTCITTCTTITGIGTG | 1 |
| 5 | 8-A PN 34 | ATGGACACICAGCTTGGTGAC | 1 |
|  | 8-A PN 124 | GCCTCAGCCACATCAAGG | 4 |
| 6 | 8-A PN 51 | CCACAGATCAAGGTGTATGC | 1 |
|  | 11-F PN 41 | CGGGGTCAGCTGACTCACC | This work |
| 7 | 11-F PN 29 | GCIGCIGACTACGCITACACYGC | 3,4 |
|  | 11-F PN 28 | GGGCCCAGGGTTGGACTC | 3 |
| 8 | 9-F PN 24 | AGGGCCGAAACATACTGCC | 4 |
|  | 8-A PN 45 | GGAAGAAACTCGAGGCGAC | 1,4 |
| 9 | 8-A PN 22 | AAGGACCCIGTCCTTGTGGC | 1,4 |
|  | 8-A PN 23 | CCGTCIAAGTGGTCAGGGTC | 1 |
| 10 | 8-A PN 46 | TGGTCGTTTGCCTCCGTGG | 1,4 |
|  | 8-A PN 87 | CTCAAAGAATTCAATTGCTGC | 1,4 |
| 11 | 8-A PN 99 | TGTACCAICTTGTTIAIGAGGTG | 1 |
|  | 9-X PN 9 | CTTTIAGAGGTITCTGACGCTC | This work |
| 12 | 8-A PN 113 | CGCGAIACTCGCAAGAGAC | 1,4 |
|  | 8-A PN 14 | ATCTCIAACTCAAACACTCTG | 1 |
| 13 | 8-A PN 80 | GGAGTGTTTGGCACTGCC | 1 |
|  | 9-X PN 16 | GCGGGTCCTTGTTGGACA | This work |
| 14 | 8-A PN 100 | CCACACCATGAAGGGTTG | This work |
|  | 8-A PN 79 | TGGGTTGCAACCGACCGC | 4 |
| 15 | 8-A PN 17 | CTGAAGGACGAIITICGICC | 4 |
|  | 8-A PN 52 | GGAITGACCAAGAACAAAACC | 4 |
| 16 | 1-A TBR 1 E | GAGCTGGACACTTACACCATGATCTC | This work |
|  | NVT27 | TTTTTTTTTTTTTTTTTTTTTTTTTTTVN | This work |

**Table S2 References**

**1:** Balinda SN, Siegismund HR, Muwanika VB, Sangula AK, Masembe C, et al (2010) Phylogenetic analyses of the polyprotein coding sequences of serotype O foot-and-mouth disease viruses in East Africa: evidence for interserotypic recombination. Virol J 7: 199.

**2:** Cottam EM, Wadsworth J, Shaw AE, Rowlands RJ, Goatley L, et al. (2008) Transmission pathways of foot-and-mouth disease virus in the United Kingdom in 2007. PLoS Pathog. 2008 Apr 18;4(4):e1000050.

**3**: Jamal SM, Ferrari G, Ahmed S, Normann P, Belsham GJ (2011) Genetic diversity of foot-and-mouth disease virus serotype O in Pakistan and Afghanistan, 1997-2009. Infect Genet Evol 11: 1229-1238.

**4.** Jamal SM, Ferrari G, Ahmed S, Normann P, Belsham GJ (2011) Molecular characterization of serotype Asia-1 foot-and-mouth disease viruses in Pakistan and Afghanistan; emergence of a new genetic group and evidence for a novel recombinant virus. Infect Genet Evol 11: 2049-2062.
